# Analysis of inflammatory immune correlation of the coagulation spectrum, PT, FBG, FDP, D-D in autoimmune diseases

**DOI:** 10.5937/jomb0-60937

**Published:** 2026-05-22

**Authors:** Yinghong Zhong, Xiao He, Xiufeng Geng, Heng Huang, Zhixian Zhang, Xuezhong Ren, Hong Chen

**Affiliations:** 1 Meizhou People's Hospital, Department of Haematology, Meizhou City, China; 2 Women and Children's Hospital Affiliated to Xiamen University Xiamen Maternal and Child Health Hospital, Department of Emergency, Xiamen City, China; 3 Jinan Blood Centre, Department of Haematology, Jinan City, China; 4 Western Theatre Command Air Force Hospital, Department of Laboratory Medicine, Chengdu City, China; 5 West China Hospital of Sichuan University, Department of Cardiology, Chengdu City, China; 6 Xinchang County People's Hospital, Department of Haematology, Shaoxing City, China

**Keywords:** international normalised ratio (INR), activated partial thromboplastin time (APTT), fibrinogen degradation products (FDP), D-dimer (D-D), autoimmune diseases, internacionalni normalizovani odnos (INR), aktivirano parcijalno tromboplastinsko vreme (APTT), proizvodi degradacije fibrinogena (FDP), D-dimer (D-D), autoimune bolesti

## Abstract

**Background:**

To investigate the expression of coagulation markers and their correlations with immunological function and inflammation in patients with autoimmune diseases (Sjogren's syndrome and rheumatoid arthritis).

**Methods:**

A total of 183 patients were selected for the study: 61 RA patients who made up the RA group were admitted to our hospital between December 2023 and December 2024, 61 pSS patients composed the pSS group, and 61 normal physical examinees who were in the physical examination centre of our hospital during the same period composed the control group. Baseline clinical indicators of patients in each group before treatment were collected, including the plasma prothrombin time (PT), international normalised ratio (INR), thrombin time (TT), plasma fibrinogen (FBG), partial thromboplastin time (APTT), fibrinogen degradation products (FDP), and plasma D-dimer (D-D).

**Results:**

The expression levels of Pt FBG, T T FDP and DD in the RA group, the pSS group and the normal group were significantly different. The expression levels of PT, FBG, FDP and D-D in the RA group were all greater than those in the pSS group and the control group. The expression level of PT in the pSS group was greater than that in the control group. Analysis of the receiver operating characteristic (ROC) curve showed that, in contrast to those in the normal group, the area under the curve (AUC) of PT in the RA group was 0.638, the AUC of FBG was 0.899, the AUC of FDP was 0.866, and the AUC of D-D was 0.919. The AUC of the combined diagnosis of RA by coagulation indicators was greater than that of the individual detection of each indicator. Compared with that in the normal group, the AUC of PT in the pSS group was 0.618 (P = 0.025), and the AUC of T T was 0.645 (P=0.006). The AUC of the combined diagnosis of coagulation indicators for pSS was greater than that of the individual detection of each indicator. Higher D-D in RA patients was significantly associated with higher levels of hs-CRP CCP and RF, while higher FBG was significantly associated with higher levels of hs-CRP ESR, RF and CCP Correlation analysis revealed that in the RA group, PT, INR, FBG, FDP and D-D were positively correlated with CRP and ESR. On the other hand, T T was negatively correlated with CRP and ESR. FBG, FDP and D-D in the pSS group were positively correlated with CRP and ESR. In addition, the coagulation indicators in the RA group were positively correlated with the immune indicators, whereas in the pSS group, they were partially negatively correlated, both of which were significant. In addition, the coagulation indicators in the RA group were positively correlated with the immune indicators, whereas in the pSS group, they were partially negatively correlated, both of which were significant. In patients with pSS, FBG and FDP are positively correlated with hs-CRP and A PTT and ESR are positively correlated with FBG.

**Conclusions:**

Compared with those of pSS, PT, FBG, FDP, and D-D, these markers have a greater diagnostic value for the early detection of RA and the assessment of disease severity, and can be used as critical predictive indicators for the confirmed diagnosis of RA.

## Abstract (sr)

**Uvod:**

Cilj ove studije bio je da se ispita ekspresija koagulacionih markera i njihova povezanost sa imunološkomfunkcijom i inflamacijom kod pacijenata sa autoimunimbolestima, uključujud Sjogrenov sindrom (pSS) i reumatoidni artritis (RA).

**Metode:**

U studiju je uključeno ukupno 183 ispitanika: 61pacijent sa RA (RA grupa) hospitalizovan u našoj bolnici uperiodu od decembra 2023. do decembra 2024. godine,61 pacijent sa primarnim Sjogrenovim sindromom (pSSgrupa), i 61 zdrav ispitanik iz centra za preventivne preglede naše bolnice, koji je činila kontrolna grupa. Od svihispitanika su prikupljeni osnovni klinički parametri prepočetka terapije, uključujuci plazmatsko protrombinskovreme (PT), internacionalni normalizovani odnos (INR),trombinsko vreme (TT), koncentraciju plazmatskog fibrinogena (FBG), aktivirano parcijalno tromboplastinsko vreme(APTT), produkte degradacije fibrinogena (FDP) i nivoplazmatskog D-dimera (D-D).

**Rezultati:**

Nivoi PT, FBG, TT, FDP i D-D bili su značajnorazličiti između RA grupe, pSS grupe i kontrolne grupe. Kod pacijenata sa RA nivoi PT, FBG, FDP i D-D bili su višinego kod pSS i kontrolne grupe, dok je nivo PT u pSSgrupi bio viši nego u kontrolnoj grupi. Analiza ROC krivepokazala je da je, u poređenju sa kontrolnom grupom,površina ispod krive (AUC) za PT u RA grupi iznosila0,638, za FBG 0,899, za FDP 0,866, a za D-D 0,919.Kombinovana dijagnostika RA korišćenjem koagulacionihindikatora dala je veću AUC vrednost od pojedinačnihindikatora. Za pSS grupu, AUC za PT bio je 0,618(P = 0 ,0 25), a za T T 0,645 (P = 0 ,0 06), pri čemu je kombinovana dijagnostika koagulacionih markera dala većudijagnostičku vrednost od pojedinačnih parametara.Povišeni D-D kod pacijenata sa RA bio je značajnopovezan sa višim vrednostima hs-CRP CCP i RF, dok jepovišeni FBG bio značajno povezan sa višim vrednostimahs-CRP, ESR, RF i CCP Korelaciona analiza pokazala je dasu u RA grupi PT, INR, FBG, FDP i D-D pozitivnokorelisani sa CRP i ESR, dok je T T negativno korelisan saCRP i ESR. U pSS grupi, fBg, FDP i D-D pozitivno sukorelisani sa CRP i ESR. Pored toga, koagulacioni indikatori u RA grupi pozitivno su korelisani sa imunološkimindikatorima, dok su kod pSS pacijenata postojale delimične negativne korelacije, što je bilo statistički značajno.Takođe, kod pSS pacijenata FBG i FDP pozitivno sukorelisani sa hs-CRP dok su A P TT i ESR pozitivnokorelisani sa FBG.

**Zaključak:**

U poređenju sa pSS, PT, FBG, FDP i D-D imajuveću dijagnostičku vrednost za ranu detekciju RA i procenutežine bolesti, te mogu služiti kao ključni prediktivni markeriza potvrđenu dijagnozu RA.

## Introduction

The pathological process of rheumatoid arthritis (RA) can involve other organs of the body, leading to systemic inflammatory responses and multiple organ involvement [1][2]. Primary Sjogren's syndrome (pSS) is a chronic autoimmune disease that primarily affects the lacrimal and salivary glands, but can also impact other organs, including the lungs, liver, pancreas, kidneys, and nervous system. However, compared with those of RA, the joint symptoms of pSS are milder, and the rate and degree of joint deformity are usually lower than those of RA [3]. Although the clinical manifestations and target organ damage of RA and pSS differ significantly, both are driven by chronic inflammation and immune dysregulation as core factors. Moreover, recent studies suggest that the activation of the coagulation system may jointly participate in disease progression through mediating vascular injury, inflammatory amplification and other pathways [4]. However, the heterogeneity of abnormal coagulation function between the two remains unclear: RA is characterised by the formation of synovial pannus and may be more likely to trigger local thrombotic inflammation; however, pSS is characterised mainly by glandular lymphocyte infiltration, and its coagulation immune interaction pattern may present different features. At present, most related studies are limited to the analysis of a single disease or a single coagulation index and lack multiparameter coagulation spectra such as the plasma prothrombin time (PT) and plasma fibrinogen (fibrinogen). A systematic comparison of FBG, fibrin (ogen) degradation products (FDPs), plasma D-dimer (D-D), etc., revealed that the value of coagulation indicators in the differential diagnosis of these two diseases remains controversial. The synergistic regulatory mechanism of the coagulation, inflammation, and immune networks also needs to be explored in depth. Therefore, systematically comparing the characteristics of abnormal coagulation function between RA patients and pSS patients not only helps to analyse the standard rules and disease-specific patterns of coagulation imbalance in autoimmune diseases but also provides a breakthrough for exploring disease classification, differential diagnosis and targeted treatment by revealing the differential associations between coagulation indicators and inflammatory and immune markers.

This study analysed the differences in coagulation indicators among RA patients, pSS patients, and healthy individuals, and innovatively combined receiver operating characteristic (ROC) curves, association rules, and multivariate regression models. The correlations between aberrant coagulation and combinations of coagulation indicators with differential diagnostic value were screened. The results not only provide a novel biomarker combination for the early differentiation of RA and pSS but also reveal the pathological mechanism of disease heterogeneity from the perspective of coagulation immune interactions, providing a theoretical basis for the development of individualised treatment strategies targeting the coagulation pathway.

## Materials and methods

### Analysis of general clinical material

Sixty-one RA patients (9 men and 52 women, ages 59.4±8.8 years on average) were admitted to our hospital's rheumatology department between December 2023 and December 2024. The categorisation criteria of ACR/EULAR. A total of 61 hospitalised patients with pSS (3 men and 58 women, ages 56.0±10.8 years on average). International Diagnostic Criteria for Primary Sjogren's Syndrome (AECG). The control group consisted of 61 normal physical examinees, chosen at random over the same time period (8 males and 53 females, with an average age of 59.1 ±10.7 years). Age and sex differences across the three groups were not statistically significant (P>0.05).

This study was reviewed and approved by the Medical Clinical Research Ethics Committee (No. HKYS-2025-A0156).

### Inclusion criteria and exclusion criteria

Inclusion criteria: (1) The RA group complied with the 2020 ACR/EUlAr classification standards, whereas the pSS group all met the international diagnostic criteria for primary Sjogren's syndrome established by the AECG in 2002 [4][5]; (2) The normal group had no immune system or major underlying diseases; and (3) the age was 18 years.

Exclusion criteria: (1) Pregnant or lactating women; (2) Those with neurological or mental disorders who were unable to cooperate; (3) Those with severe diseases such as those of the circulatory, respiratory, hematopoietic system or tumours; (4) Those with other concurrent immune system diseases.

### Laboratory testing methods and principles

Fasting venous blood was collected from the research subjects (RA group, pSS group, and healthy control group) in 109 mmol/L sodium citrate anticoagulant tubes (blood: anticoagulant = 9:1), mixed well, and then centrifuged at 3000 rpm for 15 minutes within 2 hours to obtain platelet-depleted plasma (PPP).

(1) Prothrombin Time (PT): It is based on the principle of Clotting. Add an excess of tissue thrombin (from rabbit brain or recombinant source) and calcium ion (Ca[ ) solution to the plasma sample to be tested to initiate the exogenous coagulation pathway. Instruments (Sysmex CS-5100) continuously monitor changes in plasma turbidity or viscosity through Optical Detection or Mechanical Detection.

(2) Fibrinogen (FBG): The Clauss Method is adopted, which is a derivative application of the coagulation method. Add an excess of standardised Thrombin to the plasma to be tested that has been highly diluted (to eliminate the interference of inhibitors). Thrombin directly acts on fibrinogen, converting it into fibrin monomers and polymerising them into clots. The instrument also monitors the solidification time (in seconds). The actual concentration (g/L) of fibrinogen in plasma was calculated by comparing the coagulation time with the standard curve drawn from fibrinogen standards of known concentrations.

(3) Fibrinogen Degradation Products (FDP): Latex Particle Enhanced Immunoturbidimetric Assay is adopted. Latex particles coated with specific antihuman FDP monoclonal antibodies combine with FDP antigens in the plasma to be tested, forming antigen-antibody-latex particle complexes. This complex causes a significant increase in the turbidity of the reaction solution, resulting in a decrease in the intensity of transmitted light at specific wavelengths (typically around 570 nm or 600 nm) (i.e., an increase in absorbance). The instrument detects the rate of turbidity change or the final value and compares it with the standard curve to quantitatively obtain the FDP concentration (μg/mL).

(4) D-Dimer (D-Dimer, D-D): The latex-enhanced immunoturbidimetric method is also used, and the principle is similar to that of FDP detection. The key difference lies in the fact that the latex particles are coated with monoclonal antibodies that specifically recognise the unique antigenic epitopes of the D-dimer (which only exist in the degradation products of cross-linked fibrin). This antibody only binds to the D-D fragments produced by the degradation of cross-linked fibrin (i.e., after thrombosis) by plasmin. It does not bind to the non-cross-linked fibrinogen degradation products (FDP). Measuring the changes in turbidity caused by antigen-antibody reactions and quantitatively detecting the concentration of D-D in plasma (μg/mL FEU or ng/mL DDU) is a specific marker that reflects secondary hyperfibrinolysis and thrombosis.

### Determination of laboratory indicators

The ESR was detected via Wei's method. The Hitachi 7600-020 completely automatic biochemical analyser was utilised to identify rheumatoid factor and high-sensitivity C-reactive protein (hs-CRP).

BD Vacutainer® sodium citrate Anticoagulant tube (Becton Dickinson, item number: 369714), strictly draw blood at a ratio of 9:1. Plasma was prepared using an Eppendorf Centrifuge at 5804R (3000 rpm for 15 minutes, room temperature) to obtain PPP PT: Thromborel S (Siemens Healthineers, item No. OWRU2153); FBG: Multifibren U (Siemens Healthineers, item No. OWRH2153) (Clauss method); FdP/D-D: LPIA Kit (Sysmex, FDP catalogue number: 0010007411; D-D catalogue number: 0010007412) (Latex immunoturbidimetric method).

### Statistical analysis

Statistical analysis was conducted using SPSS 27.0, SPSS Modeller 18.0, and GraphPad Prism 10 software. The measurement data conforming to a normal distribution are expressed as x̄±s and x <e:1> ± s. M (P_25_, P_75_) is used to represent measurement data that do not fit a normal distribution. The chi-square test was used for count data. A P value < 0.05 was considered statistically significant.

## Results

### Comparison of the expression levels of coagulation indicators in the RA, pSS and control groups

PT, FBG, TT, FDP and DD among the RA group, the pSS group and the normal group (P<0.05), whereas there was no statistically significant difference in the INR or APTT (P >0.05). PT, FBG, FDP and D-D expression levels were considerably higher in the RA group than in the PSS group and the control group (P<0.05 or P<0.01). In the PSS group, a statistically significant difference was observed in the expression level of PT compared to the control group (P<0.05, Table 1).

**Table 1:** Comparison of coagulation indicators among RA group, pSS group and Normal Group (n = 61, x̄ ± s, M (P25, P75).

Indicator	RA group	pSS group	Normal group	H/x^2^
TT (s)	18.61±0.87	18.24±1.36	18.89±1.31	4.454
PT (s)	10.70 (10.00, 11.50)	10.60 (10.20, 11.10)	10.40 (10.00, 10.70)a	8.343
INR	0.9 5(0.90, 1.00)	0.95 (0.91, 0.99)	0.94 (0.92, 0.98)	0.193
APTT (s)	28.70 (27.10, 30.30)	29.10 (27.80, 30.80)	28.80 (27.60, 30.00)	2.399
FBG (g/L)	4.58 (3.66, 5.38)d	2.79 (2.48, 3.24)	2.80 (2.47, 3.22)	71.594
FDP (mg/L)	2.40 (1.50, 3.30)	0.70 (0.60, 1.00)	0.70 (0.60, 1.00)	65.082
D-D (mg/L)	1.73 (0.86, 2.38)	0.25 (0.19, 0.48)	0.28 (0.19, 0.39)	76.844

### Analysis of the six coagulation parameters using receiver operating characteristic curves to estimate RA and pSS

The best cutoff time was 0.528 seconds, the sensitivity and specificity for predicting RA were 0.426 and 0.918, respectively, and the area under the ROC curve of PT in the RA group was 0.638 (P=0.009, P<0.01). The sensitivity and specificity for RA prediction were 0.745 and 0.967, respectively, and the best cutoff value was 0.610 g/L. The area under the ROC curve of FBG was 0.899. The optimal cutoff value was 0.341 μg/mL, yielding sensitivity and specificity of 0.836 and 0.803, respectively, for RA prediction, and an area under the ROC curve of 0.866 for FDP The best cutoff value was 0.441 μg/mL, the sensitivity and specificity for RA prediction were 0.803 and 0.984, respectively, and the area under the ROC curve of D-D was 0.919. With an ideal cutoff value of 0.691, the area under the combined ROC curve of PT, INR, APTT, FBG, TT, FDP and D-D was 0.985. The results showed that PT, FBG, FDP, and D-D have some predictive efficacy for the diagnosis of RA, with sensitivities and specificities for RA prediction of 0.934 and 0.984, respectively (Table 2).

**Table 2:** Relevant parameters of coagulation indicators for the diagnostic efficacy of RA.

Indicator	Cutoff value	Sensitivity	Specificity	AUC	95%CI	P
PT (s)	0.582	0.426	0.918	0.638	0.537-0.739	0.007
INR	0.521	0.311	0.902	0.522	0.415-0.629	0.685
APTT (s)	0.524	0.344	0.787	0.534	0.431-0.638	0.515
FBG (g/L)	0.610 g/L	0.754	0.967	0.899	0.841-0.958	<0.001
TT (s)	0.448 sec	0.934	0.344	0.59	0.486-0.694	0.089
FDP (mg/L)	0.341 μg/mL	0.836	0.803	0.866	0.800-0.932	<0.001
D-D (mg/L)	0.441 mg/L	0.803	0.984	0.919	0.867-0.971	<0.001
PT+INR+APTT+FBG+TT+<br>FDP+D-D	0.691	0.934	0.984	0.985	0.968-1.002	<0.001

The PSS group's PT area under the ROC curve was 0.618 (P=0.025, P<0.05), the TT area under the ROC curve was 0.645 (P=0.006), the optimal cutoff value was 0.585 seconds, and the sensitivity and specificity for predicting pSS were 0.344 and 0.918, respectively. The optimal cutoff value was 0.493 sec, and the sensitivity and specificity for predicting pSS were 0.672 and 0.623, respectively. The area under the combined ROC curve of PT, INR, APTT, FBG, TT, FDP and D-D was 0.985, and the optimal cutoff value was 0.480. The sensitivity and specificity for predicting pSS were 0.721 and 0.656, respectively, indicating that the individual indicators PT and TT have limited predictive value. Combined detection can assist in diagnosis, but its efficacy is significantly lower than that of RA (Table 3).

**Table 3:** Relevant parameters of coagulation indicators for the diagnostic efficacy of pSS.

Indicator	Cutoff value	Sensitivity	Specificity	AUC	95%CI	P
PT (s)	0.585	0.344	0.918	0.618	0.518-0.718	0.021
INR	0.526	0.131	0.984	0.508	0.404-0.612	0.875
APTT (s)	0.538	0.279	0.885	0.548	0.445-0.650	0.364
FBG (g/L)	0.567	0.164	0.984	0.510	0.407-0.614	0.847
TT (s)	0.493	0.672	0.623	0.645	0.546-0.744	0.004
FDP (mg/L)	0.498	0.492	0.574	0.504	0.401-0.602	0.939
D-D (mg/L)	0.566	0.230	0.984	0.506	0.401-0.612	0.905
PT+INR+APTT+FBG +<br>TT+FDP+D-D	0.480	0.721	0.656	0.985	0.968-0	<0.001

### Association analysis of coagulation indicators with inflammatory factors and immune indicators in RA patients and pSS patients

For RA patients, D-D and FBG are used as the preceding items, and inflammatory factors and immune indicators are used as the subsequent items, with a support degree of >30%, a confidence degree of >70%, and an enhancement degree of >1. An analysis of the Apriori algorithm revealed that an increase in D-D was strongly associated with increases in hs-CRP CCP and RF An increase in FBG is strongly correlated with increases in hs-CRP ESR, RF and CCP For patients with pSS, D-D and the international normalised ratio (INR) were used as the preceding items, and inflammatory factors and immune indicators were used as the subsequent items, with support degrees >10%, confidence degrees >50%, and enhancement degrees >1. Through the analysis of the Apriori algorithm, the increase in D-D is associated to a certain extent with the rise in hs-CRP and anti-SSA. An increase in the INR is associated to some extent with increases in hs-CRP, anti-SSA and anti-SSB. The correlation analyses among the remaining indicators all failed to reach the set threshold, and no results were produced. The research showed that the RA group's connections with inflammatory variables, immunological indicators, and coagulation indicators were more pronounced than those in the pSS group (Table 4 and Table 5).

**Table 4:** Analysis of association rules between coagulation indicators and inflammatory factors in RA.

Preceding item	The following item	Support rate (%)	Confidence level (%)	Degree of improvement
D-D↑ (mg/L)	RF↑	79.032	97.959	1.047
D-D↑ (mg/L)	hs-CRP↑	79.032	95.918	1.062
D-D↑ (mg/L)	CCP↑	79.032	87.755	1.110
FBG↑ (g/L)	RF↑	66.129	97.561	1.043
FBG↑ (g/L)	hs-CRP↑	66.129	97.561	1.080
FBG↑ (g/L)	CCP↑	66.129	85.366	1.080
FBG↑ (g/L)	ESR↑	66.129	73.171	1.418

**Table 5:** Analysis of association rules between coagulation indicators and inflammatory factors in pSS.

The preceding item	The following item	Support rate (%)	Confidence level (%)	Degree of improvement
D-D↑ (mg/L)	hs-CRP↑	21.311	76.923	1.466
D-D↑ (mg/L)	SSA↑	21.311	61.538	0.751
INR↑	hs-CRP↑	13.115	75.000	1.430
INR↑	SSA↑	13.115	87.500	1.068
INR↑	SSB↑	13.115	50.000	1.173

### Correlation analysis of the six coagulation parameters with RA, pSS inflammation and disease indicators

Through correlation analysis of inflammatory and immune indicators and coagulation indicators, CRP in the RA group was found to be positively correlated with PT, INR, FBG, TT, FDP and D-D (r=0.3681, r=0.3793, r=0.6363, r=0.2532, r=0.4562, r=0.3842) (P=0.0021, P=0.0026, P<0.0001, P=0.0489, P=0.0002, P=0.0022). ESR was positively correlated with PT, INR, FBG, TT, FDP and D-D (r=0.4296, r=0.4831, r=0.7487) r=0.3066, r=0.5198, r=0.4792, P=0.0005, P<0.0001, P<0.0001, P=0.0163, P<0.0001, P<0.0001; IgG was positively correlated with PT, INR, APTT, and TT (r=0.412, r=0.465, P (P=0.031, P=0.018), and PT, the INR, and APTT showed favorable correlations with IgG4 (r=0.374, r=0.467, r=0.369, P=0.003, P<0.001, P=0.004). A statistically significant association was found (P<0.05, Table 6).

**Table 6:** Correlation analysis of six coagulation indicators and RA disease-related indicators.

Indicator	PT		INR		APTT		FBG		TT		FDP		D-D	
	rs	P	rs	P	rs	P	rs	P	rs	P	rs	P	rs	P
CRP (mg/L)	0.389	0.002	0.379	0.003	0.190	0.142	0.636	<0.001	-0.253	0.049	0.456	<0.001	0.384	0.002
ESR (mm/h)	0.430	0.001	0.483	<0.001	0.185	0.154	0.776	<0.001	-0.307	0.016	0.512	<0.001	0.479	<0.001
RF (IU/mL)	0.008	0.948	0.129	0.320	-0.245	0.057	0.232	0.072	-0.201	0.120	0.175	0.177	0.158	0.224
CCP (U/mL)	0.102	0.436	0.170	0.190	-0.099	0.447	0.161	0.215	-0.082	0.528	0.165	0.203	0.220	0.088
C3 (g/L)	0.066	0.613	0.003	0.982	0.147	0.258	0.582	<0.001	-0.106	0.417	0.203	0.117	0.246	0.056
C4 (g/L)	-0.034	0.798	-0.015	0.909	0.275	0.032	0.207	0.108	-0.027	0.836	-0.054	0.682	-0.024	0.856
IgG (g/L)	0.412	0.001	0.465	<0.001	0.416	<0.001	-0.088	0.500	0.275	0.032	-0.041	0.751	-0.082	0.531
IgM (g/L)	0.276	0.031	0.301	0.018	0.231	0.074	0.073	0.577	-0.057	0.661	0.242	0.061	0.223	0.085
IgG (g/L)	0.374	0.003	0.467	<0.001	0.369	0.004	0.143	0.271	-0.023	0.863	0.161	0.214	0.065	0.618

CRP in the PSS group was found to have a positive correlation with FBG, FDP, and D-D through correlation analysis of coagulation indicators and inflammatory and immunological markers in patients with pSS (r = 0.658, r = 0.470, r = 0.450, P < 0.001, P < 0.001, P < 0.001). FBG, FDP and D-D showed positive correlations with the ESR (r = 0.669, r=0.508, r=0.448, P<0.001, P<0.001, P<0.001), whereas C3 was negatively correlated with PT and the INR (r=-0.331, r=-0.286, P=0.009, P=0.026). C3 was positively correlated with FDP and D-D (r=0.352, r=0.272, P=0.005, P= 0.034), C4 was negatively correlated with the INR (r=-0.300, P=0.019), and C4 was positively correlated with FDP (r=0.311, P=0.015) (Table 7).

**Table 7:** Correlation analysis of six coagulation indicators and disease-related indicators of PSS.

Indicator	PT		INR		APTT		FBG		TT		FDP		D-D	
	rs	P	rs	P	rs	P	rs	P	rs	P	rs	P	rs	P
CRP<br>(mg/L)	-0.182	0.159	-0.111	0.395	-0.147	0.258	0.658	<0.001	-0.031	0.814	0.470	<0.001	0.450	<0.001
ESR<br>(mm/h)	0.054	0.681	0.144	0.269	0.167	0.199	0.669	<0.001	-0.117	0.368	0.508	<0.001	0.448	<0.001
RF<br>(IU/mL)	0.084	0.525	0.141	0.283	0.034	0.797	0.137	0.298	-0.014	0.917	-0.067	0.612	-0.099	0.450
CCP<br>(U/mL)	0.193	0.137	0.185	0.152	-0.001	0.997	0.071	0.586	0.121	0.355	0.047	0.718	0.047	0.721
C3 (g/L)	-0.174	0.180	0.330	0.019	-0.193	0.136	0.225	0.082	0.127	0.329	0.311	0.015	0.163	0.209
C4 (g/L)	0.140	0.282	0.255	0.048	0.158	0.224	0.202	0.118	0.225	0.082	0.181	0.162	0.176	0.176
IgG (g/L)	0.038	0.770	0.134	0.304	0.154	0.236	0.342	0.007	0.051	0.698	0.241	0.062	0.245	0.058
IgA (g/L)	0.028	0.832	0.150	0.248	0.128	0.325	0.273	0.033	0.094	0.473	0.159	0.222	0.053	0.684
IgM (g/L)	0.225	0.081	0.238	0.065	0.150	0.249	0.015	0.908	0.145	0.265	0.073	0.575	0.023	0.861
anti-SSA<br>(U/mL)	0.067	0.610	0.025	0.851	0.110	0.399	0.033	0.802	0.113	0.387	0.216	0.095	0.262	0.042

### Multivariate linear regression analysis of hs-CRP and ESR levels in patients with RA and pSS

**Figure 1:**
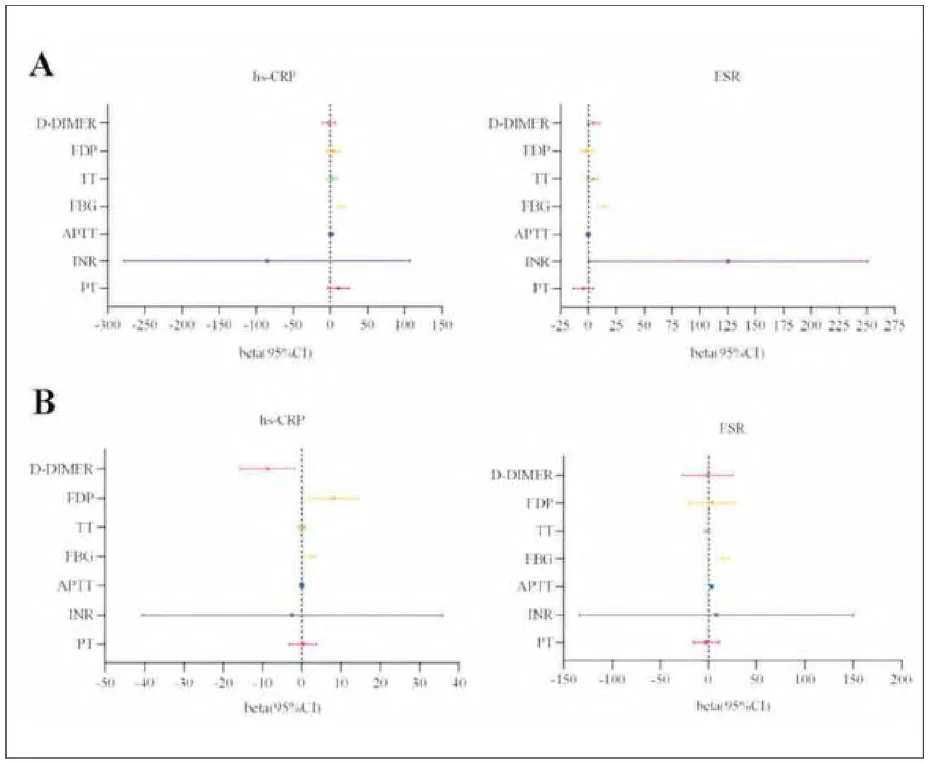
Influencing factors of hs-CRP and ESR in patients with RA and pSS.<br>A: Forest plot of the factors influencing the hs-CRP level and ESR in RA patients. B: Forest plot of the factors influencing the hs-CRP level and ESR in pSS patients.

The levels of hs-CRP and ESR in RA patients and pSS patients were used as dependent variables, while coagulation indicators such as PT, APTT, TT, FBG, FDP and D-D were used as independent variables (P<0.05, Table 8 and Table 9). In patients with pSS, FBG and FDP are positively correlated with hs-CRP and APTT and ESR are positively correlated with FBG (Figure 1). Therefore, abnormal coagulation functions (FBG, FDP, APTT) are closely related to the inflammatory activities of RA and pSS; however, the specific association patterns reveal disease heterogeneity.

**Table 8:** Multiple linear regression analysis affecting the levels of hs-CRP and ESR in RA patients.

Indicator	hs-CRP			ESR		
	B	P	95%CI	B	P	95%CI
PT (s)	11.33	0.132	-3.203~25.863	-4.368	0.368	-13.803~5.066
INR	-85.683	0.387	-278.251~106.884	125.552	0.054	0.540~250.563
APTT (s)	1.056	0.449	-1.660~3.772	-0.133	0.883	-1.896~1.630
FBG (g/L)	13.328	0.001	6.295~20.362	13.381	0.001	8.814~17.947
TT (s)	2.763	0.469	-4.658~10.184	3.79	0.129	-1.027~8.608
FDP (mg/L)	3.802	0.4	-4.972~12.576	-1.653	0.572	-7.349~4.043
D-D (mg/L)	-2.666	0.539	-11.114~.782	4.566	0.109	-0.918~10.050

**Table 9:** Multiple linear regression analysis affecting the levels of hs-CRP and ESR in patients with PSS.

Indicator	hs-CRP			ESR		
	B	P	95%CI	B	P	95%CI
PT (s)	0.274	0.881	-3.308~3.856	-2.316	0.735	-15.669~11.038
INR	-2.473	0.9	-40.684~35.737	8.398	0.908	-134.044~150.840
APTT (s)	0	0.999	-0.405~0.405	3.374	0.001	1.864~4.884
FBG (g/L)	2.297	0.010	0.611~3.984	16.096	0.001	9.810~22.381
TT (s)	0.191	0.695	-0.757~1.139	-1.264	0.486	-4.797~2.268
FDP (mg/L)	8.106	0.017	1.657~14.554	3.98	0.747	-20.058~28.019
D-D (mg/L)	-8.666	0.019	-15.681~-1.652	-0.563	0.966	-26.711~25.585

## Discussion

Both RA and pSS are chronic systemic autoimmune diseases that require long-term management. The pathological core of RA is synovitis, which can involve multiple organs. pSS originates from lymphocyte infiltration of the exocrine glands [1][3]. The ESR is a dynamic indicator of RA and pSS. It can drop to normal when inflammation is stably controlled and is used to assess the degree of inflammation [6][7]. CRP is a specific and sensitive marker for the early diagnosis of RA and pSS, and is less affected by sex, age, and plasma protein levels. It can be used to track disease activity and evaluate the effects of treatment [8][9]. In this study, coagulation indicators were combined with the ESR and CRP for analysis to explore their diagnostic and predictive efficacy. As a specific serum marker for RA, the anti-CCP antibody is crucial for RA diagnosis but of little use in pSS diagnosis. RF has a high positive rate in RA and is a key indicator for RA diagnosis. However, it can also be positive in patients with pSS, which is prone to misdiagnosis. Moreover, it has particular predictive value for the salivary glands and extramandular organs in pSS patients [10]. Although they have a high positive rate for pSS, anti-SSB and anti-ssa antibodies are not diagnostically significant for RA [11]. Therefore, in this study, anti-CCP antibodies and RF were used as RA indicators, anti-SSA antibodies and anti-SSB antibodies were used as pSS indicators, and an association analysis was conducted with coagulation indicators.

This finding is consistent with the pathophysiological characteristics of RA. The continuous inflammatory response in RA patients leads to an imbalance in the coagulation and anticoagulation systems. The pathological process not only triggers coagulation but also disrupts the natural anticoagulation pathway, slows fibrinolytic activity, and is often accompanied by vascular endothelial injury, platelet hyperactivity, disorders of the coagulation and fibrinolytic systems, and abnormal blood microcirculation, all of which indicate a hypercoagulable blood environment [12]. PT reflects the activity of coagulation factors VII, X, V, II, and I (fibrinogens). ROC curve analysis indicated that PT, FBG, FDP and D-D had high diagnostic value in RA patients. The areas under the ROC curve were all greater than 0.6, with D-D being the highest, at 0.919, demonstrating good diagnostic efficacy. D-D, a key indicator of the process of thrombosis formation and dissolution, is a specific degradation product of fibrin. An elevated level of D-D usually indicates the formation and degradation of fibrin in the body and is a sensitive indicator of the state of thrombosis formation and dissolution. Its increase may reflect enhanced coagulation and inflammatory activities in RA patients [13][14].

Increased inflammatory and disease markers, including hs-CRP CCP and RF, were substantially correlated with elevated D-D levels in RA patients, according to further association rule analysis. In contrast, elevated FBG was associated with increased indicators, such as hs-CRP ESR, RF, and CCP These findings further confirm the close connection between the coagulation function of RA patients and inflammatory activity as well as disease indicators. These results may reflect the connection between abnormal coagulation function, inflammation, and the immune response in pSS patients [15][16].

This study revealed that in RA patients, the PT and INR were positively correlated with the CRP level and ESR, indicating that the activation of coagulation function was associated with increased inflammatory activity. The strong positive correlation between FBG, CRP and ESR further highlights the close connection between the coagulation cascade and inflammation [17]. The positive correlations of FDP and D-D with CRP and ESR indicate that increased fibrinolysis in RA is associated with increased inflammatory activity. The negative correlation between TT, CRP and ESR may suggest that in an inflammatory environment, prolonged TT is associated with coagulation inhibition, which might be a regulatory mechanism of the body's response to inflammation [18]. In patients with pSS, the positive correlation between FBG or CRP and the ESR indicates that fibrinogen levels are associated with increased inflammatory activity. The positive correlations between FDP and D-D and between CRP and the ESR further confirmed the connection between inflammation and coagulation activation in pSS patients [19][2][20]. In RA, the positive correlation between C3 and FBG may reflect the role of the complement system in regulating the coagulation cascade. In pSS, the negative correlation between C3 and the PT and INR may indicate that complement system activation is associated with the inhibition of coagulation function, which may be a pathophysiological feature of pSS [21][22][23][24].

Multiple linear regression analysis further confirmed the specific associations between coagulation indicators and inflammatory activity. In RA patients, FBG positively regulates the levels of hs-CRP and ESR, which is consistent with its dual function as an acute-phase response protein. It not only aggravates synovial inflammation through fibrin deposition but also forms an »inflammation coagulation« positive feedback loop by activating endothelial cells and proinflammatory factors such as IL-6 [25][26][27]. This finding is corroborated by the significant increase in FBG in the RA group, as well as its strong positive correlation with CRP and ESR, suggesting that FBG can serve as a key bridge between inflammatory activity and thrombosis risk in RA patients. The coagulation inflammation association in patients with pSS presents a unique pattern. FBG and FDP jointly positively drive hs-CRP whereas APTT and FBG jointly regulate ESR [28][29][30][31]. The prolongation of APtT may be related to the consumption of coagulation factors caused by chronic inflammation, and its positive correlation with the ESR may reflect the balance mechanism between complement activation (such as C3 inhibition of coagulation factors) and local fibrinolysis enhancement in pSS patients [32][33][34].

## Conclusion

The expression levels of coagulation indicators (especially PT, FBG, FDP and D-D) are significantly elevated in RA patients and are closely related to inflammation and immune responses. These findings have significant reference value for the early diagnosis and severity assessment of RA. These findings suggest that coagulation indicators may have significant clinical value in the diagnosis and treatment of RA. It helps assess the inflammatory activity and thrombosis risk of RA patients.

## Dodatak

### Authors' contributions

The first authors of the study are Yinghong Zhong and Xiao He.

### Conflict of interest statement

All the authors declare that they have no conflict of interest in this work.
